# Numerical Energy Storage Efficiency of MWCNTs-Propylene Glycol by Inducing Thermal Radiations and Combined Convection Effects in the Constitutive Model

**DOI:** 10.3389/fchem.2022.879276

**Published:** 2022-05-30

**Authors:** Adnan, Waqas Ashraf, Ilyas Khan, Mohamed A. Shemseldin, Abd Allah A. Mousa

**Affiliations:** ^1^ Department of Mathematics, Mohi-ud-Din Islamic University, Trarkhel, Pakistan; ^2^ Departmment of Applied Mathematics and Statistics, Institute of Space Technology (IST), Islamabad, Pakistan; ^3^ Department of Mathematics, College of Science Al-Zulfi, Majmaah University, Al-Majmaah, Saudi Arabia; ^4^ Mechanical Engineering Department, Faculty of Engineering and Technology, Future University in Egypt, New Cairo, Egypt; ^5^ Department of Mathematics and Statistics, College of Science, Taif University, Taif, Saudi Arabia

**Keywords:** MWCNTs-PG, thermal radiations, combined convection, thermal storage, nanofluid empirical correlations

## Abstract

This study examines MWCNTs-PG nanofluid with a uniform dispersion of MWCNTs in PG. It is assumed that both MWCNTs and PG exist thermally in equilibrium and no slip occurs between them. MWCNTs were suspended in PG uniformly and played a significant role. Firstly, the problem is formulated by utilizing empirical correlations, thermophysical attributes, and similarity equations. Then the model is treated numerically along with the coupling of a shooting algorithm. The results against the pertinent flow quantities were plotted and provide a basis for a comprehensive discussion, investigating whether MWCNTs-PG has high thermal storage characteristics under the effects of thermal radiation and combined convection effects. Due to their high energy storage capability, these fluids are reliable for industrial applications.

## Introduction

Thermal storage is a long-standing issue for industrialists and engineers. Researchers have posited that the energy efficiency of regular liquids could be improved by dispersing nano additives of various metals or their oxides in host liquids. The common base fluids (Propylene glycol (PG) 0.147 W/mk, ethylene glycol (EG) 0.258W/mK, water, engine oil 0.145 W/mK, and kerosene oil 0.145 W/mk etc.) have limited industrial applications due to their low thermal performance. The low thermal conductivity of the aforementioned fluids means they have inadequate capacity for energy storage. They could be made more effective and applicable by dispersing nanoparticles, which upsurges the energy storage of the resultant fluid. Thus, a new sort of heat transport fluid was developed which is a colloidal suspension of the metallic nano-sized particles and the regular liquid. Due to the high thermal conductivity of the metallic particles, the internal energy of the colloidal mixture rises, which significantly alters the thermal storage ability of the mixture. These fluids are termed Nanofluids due to the addition of nano additives of the metals.

The development of nanofluids has been a popular research subject among engineers and industrialists, with potential applications in every aspect of daily life. The core applications of these fluids can be found in the manufacturing of kitchen appliances, the medical sciences, the detection of cancer cells in the human body, manufacturing air craft parts, detergents, paint industries, computer chips, civil engineering, automobile and mechanical engineering, wire coating, air buses, aerodynamics, and ceramics etc. As the modern world depends on nanotechnology, which is incredible except for nanofluids. The study of nanofluids dynamics has become a hub for researchers and engineers from all over the world, resulting in innovative research on nanofluids under various physical circumstances. Therefore, fluid dynamists have examined the behavior of energy storage in nanofluids under certain physical scenarios.

The investigation of heat and mass transport mechanism in Williamson nanoliquid over a stretchable surface was examined by Khan et al. (2018). They imposed slanted Lorentz forces with strength B_0_ in the cartesian frame and treated the resultant model by adopting a numerical technique and discussed the results deeply against the physical constraints. Another significant investigation examined the energy efficiency in the nanofluid by taking the effects of convective surface and fluid internal heat or sink (Gireesha et al., 2018). From the obtained results they concluded that nonlinear thermal radiation is a potential physical source for thermal enhancement in the nanofluids. The nanoliquid heated through nonlinear thermal radiations boosts the internal energy of the liquid particles and hence the energy storage ability of the nanoliquid goes up.

In 2016, Uddin et al. (2016) prolonged the analysis of nanofluid by taking microorganisms into account. With innovative results, they considered the influence of buoyancy forces and Stefan blowing in the nanoliquid. Lately, Punith Gowda et al. (2021) performed a computational investigation for the nanofluid by inducing KKL thermal conductance correlation to intensify the energy storage of nanofluids. The results reveal that thermal transport enhances due to stronger porosity effects while the velocity gradient declines against it. Recently, Waini et al. (2020) conducted an investigation of thermal transport in hybrid nanofluid past a stretching surface and provide a comprehensive discussion regarding the dynamics of nanofluids. The study of thermodynamics second law for nanoliquid flow through a curved geometry is reported by Hayat et al. (2020a) in the existence of partial slip. From the results, they observed that thermal radiations and Biot effects directly affect the temperature of nanoliquid while Bejan number drops for higher slip effects.

The characteristics of the stagnation point flow by inducing the CC heat flux model in the constitutive relation for Oldroyd B-nanoliquid, as performed by Hayat et al. (2020b). The reduction in the fluid motion is noticed against retardation and relaxation time parameters. Another investigation examined the characteristics of the nanofluid prepared by ferromagnetic particles (Yasmeen et al., 2016). The influences of homogeneous and heterogeneous reactions and magnetic dipole effects are ingrained in the model, to examine effects on the temperature. Heat transport investigations of nanofluid have considered various physical parameters, as reported in several studies (Shaiq and Maraj, 2019; Benos et al., 2019; Das et al., 2015). Applied magnetic field and velocity slip effects on the dynamics of fluid flow organized over a vertically oriented surface were examined by Mukhopadhyay and Chandra Mandal (2015) in 2015. Another significant study on the presence of combined convective effects over a slippery surface was reported by Bhattacharyya et al. (2013). Furthermore, important studies regarding the numerical treatment of concentrated thermal system, solvothermal preparation of high performance, cascade nanofluid based on PV/T system, and condensation triggered synthesis of dual channels have also been reported (Yazdanifard et al., 2020; Bej et al., 2021; Hassani et al., 2016; Bej et al., 2022). The contribution of Fourier heat flux to the thermal enhancement for carbon nanotubes based on the nanofluid inside the cavity was reported by Reddy and Sreedevi (2021). Insights into entropy generation and heat transfer under the varying effects of the magnetic field were determined by another important study (Reddy et al., 2022).

This study explores the:

• Energy storage efficiency in MWCNTs-PG over an inclined surface.
• Role of thermal radiations in the heat transfer mechanism.
• Combined convection effects and their contribution to the velocity and thermal performance of MWCNTs-PG.
• The velocity and thermal slip effects on fluid movement and heat transfer over an inclined surface.

A careful literature review revealed that the energy storage efficiency in the nanofluid, namely MWCNTs-PG, has not conducted in real world applications to date. Therefore, the analysis examines this nanoliquid under the novel effects of thermal radiations and the combined convection effects on the dissipative nanoliquid. A set of similarity equations will be utilized for dimensionless mathematical modeling and then an efficient numerical algorithm based on the shooting technique will be adopted for computation of the results for motion and the thermal behavior of the nanoliquid. Moreover, the trends of the shear stresses and local thermal performance rate in MWCNTs-PG are discussed in detail.

## Development of MWCNTs-PG Model

### Problem Statement and Geometry

**FIGURE 1:**
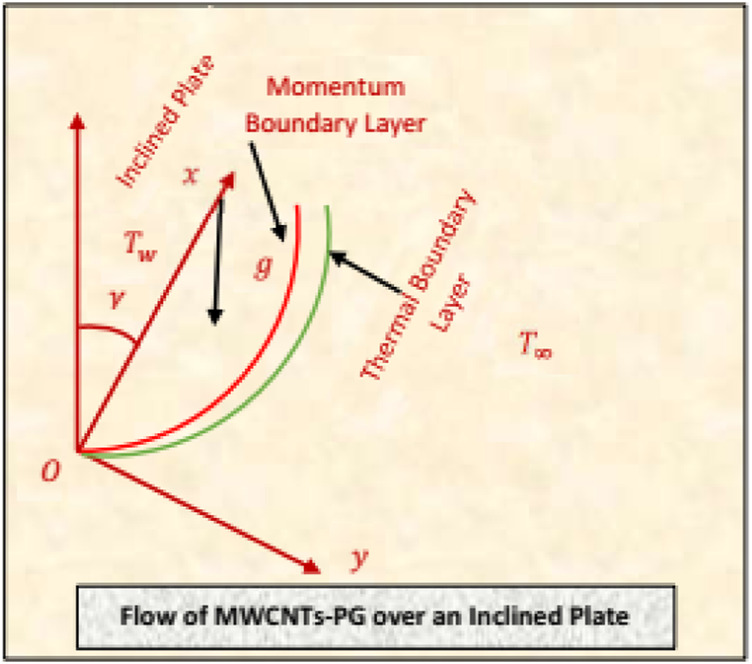
The Flow configuration of MWCNTs-PG nanoliquid.

The flow configuration of MWCNTs-PG is considered over an inclined surface positioned in cartesian coordinates. The surface is inclined at an acute angle through the vertical axis oriented in an anticlockwise direction. To examine an innovative behavior of the nanofluid, influences of combined convection plugged in the governing model. Moreover, the flow is radiative, and viscous dissipation effects are contemplated. The fluid temperature is maintained at $T_{w}$ at the surface and $T_{\infty \;}$ far from the surface. The nanofluid flows in the region y>0 . It is assumed that MWCNTs will be dispersed in PG uniformly, that they are thermally compatible, and thatno slip occurs between them. Furthermore, the velocity and thermal slips are imposed over the surface. The flowing scenario of MWCNTs-PG is depicted in Figure 1.

### Empirical Correlations and Thermophysical Attributes for MWCNTs-PG

To boost the energy storage of MWCNTs-PG, the following thermophysical attributes are ingrained in the constitutive model. These correlations are given in Table 1:

**TABLE 1:** Thermophysical attributes of the nanofluid (Ahmed et al., 2020), (Ahmed et al., 2017).

S. No	Thermophysical attributes	Mathematical correlation
1	Dynamic viscosity	${\overset{︷}{\mu }}_{nf}=\overset{︷}{\underset{f}{\mu }}{\left(1-\phi \right)}^{-2.5}$
2	Effective density	$\overset{︷}{\underset{nf}{\rho }}={\overset{︷}{\rho }}_{PG}\left\{\left(1-\phi \right)+\frac{\phi {\overset{︷}{\rho }}_{MWCNTs}}{{\overset{︷}{\rho }}_{PG}}\right\}\;$
3	Effective thermal conductance	$\overset{︷}{\underset{nf}{k}}={\overset{︷}{k}}_{PG}\left\{\frac{\left(1-\phi \right)+\frac{\frac{2\phi \overset{︷}{\underset{MWCNTs}{k}}}{\overset{︷}{\underset{MWCNTs}{k}}-\overset{︷}{\underset{PG}{k}}}\ln\left(\overset{︷}{\underset{MWCNTs}{k}}+\overset{︷}{\underset{PG}{k}}\right)}{2\overset{︷}{\underset{PG}{k}}}}{\left(1-\phi \right)+\frac{\frac{2\phi \overset{︷}{\underset{PG}{k}}}{\overset{︷}{\underset{MWCNTs}{k}}-\overset{︷}{\underset{PG}{k}}}\ln\left(\overset{︷}{\underset{MWCNTs}{k}}+\overset{︷}{\underset{PG}{k}}\right)}{2\overset{︷}{\underset{PG}{k}}}}\right\}$
4	Heat capacity	${\left(\overset{︷}{\rho c_{p}}\right)}_{nf}={\left(\overset{︷}{\rho c_{p}}\right)}_{PG}\left\{\left(1-\phi \right)+\frac{\phi {\left(\overset{︷}{\rho c_{p}}\right)}_{MWCNTs}}{{\left(\overset{︷}{\rho c_{p}}\right)}_{PG}}\right\}$

In Table 1, subscript nf stands for nanofluid and ϕ is the volumetric fraction of MWCNTs. The thermophysical values of the host liquid PG and MWCNTs are elaborated in Table 2 (Shaiq and Maraj, 2019; Benos et al., 2019):

**TABLE 2:** Thermophysical values of MWCNTs and PG.

Characteristics	H_2_O	PG	MWCNTs	SWCNTs
$\overset{︷}{\rho }\left(Kgm^{-3}\right)$	997.1	938.5	1600	2600
$\overset{︷}{\mu }\left(Pas\right)$	$0.85\times 10^{-3}$	—	—	—
Cp︷ (JKg^(−1) K^(−1))	4179	4,338	796	425
$\overset{︷}{k}\left(Wm^{-1}K^{-1}\right)$	0.613	0.684	3,000	6,600
$\overset{︷}{\beta }\left(K^{-1}\right)$	$2.1\times 10^{-4}$	—	$42\times 10^{-6}$	—
$\overset{︷}{\sigma }\left(Sm^{-1}\right)$	0.05	—	$4.8\times 10^{-7}$	—
Pr	7.2	1.458	—	—

### Constitutive Flow Model for MWCNTs-PG

Under the assumptions mentioned in the problem statement, the following relations constitute the flow of MWCNTs-PG over an inclined surface by incorporating the influences of combined convection, viscous dissipation, and thermal radiations (Das et al., 2015):

$$\frac{\partial \overset{\vee }{u}}{\partial x}+\frac{\partial \overset{\vee }{v}}{\partial y}=0,$$ (1)

$${\overset{︷}{\rho }}_{nf}\left(\overset{\vee }{u}\frac{\partial \overset{\vee }{u}}{\partial x}+\overset{\vee }{v}\frac{\partial \overset{\vee }{u}}{\partial y}\right)-{\overset{︷}{\mu }}_{nf}\frac{\partial^{2}\overset{\vee }{u}}{\partial y^{2}}-\hat{g}{\left(\overset{︷}{\rho }{\overset{︷}{\beta }}^{*}\right)}_{nf}\left(\overset{︷}{T}-{\overset{︷}{T}}_{\infty }\right)cos\gamma =0$$ (2)

$${\left(\hat{\overset{︷}{\rho C_{p}}}\right)}_{nf}\left(\overset{\vee }{u}\frac{\partial \overset{\vee }{T}}{\partial x}+\overset{\vee }{v}\frac{\partial \overset{\vee }{T}}{\partial y}+\overset{\vee }{w}\frac{\partial \overset{\vee }{T}}{\partial z}\right)-{\overset{︷}{k}}_{nf}\frac{\partial^{2}\overset{\vee }{T}}{\partial y^{2}}-{\overset{︷}{\mu }}_{nf}{\left(\frac{\partial \overset{\vee }{u}}{\partial y}\right)}^{2}+\frac{16\sigma^{*}{\hat{T}}_{\infty }^{3}}{3K^{*}}\frac{\partial^{2}\overset{\vee }{u}}{\partial y^{2}}=0$$ (3)

The flow over the surface obeys the following conditions at the surface and far from it:

$$\overset{\vee }{u}=\frac{\partial \overset{\vee }{u}}{\partial y}L,\overset{\vee }{v}=-{\overset{\vee }{u}}_{w},\overset{︷}{T}=\overset{︷}{\underset{w}{T}}+\frac{\partial \overset{\vee }{T}}{\partial y}K\;\text{at}\;y=0$$

$$\overset{\vee }{u}\to U_{\infty },\overset{︷}{T}\to \overset{︷}{\underset{\infty }{T}}\;\text{at}\;y\to \infty$$

In the above conditions, $\overset{︷}{\underset{w}{T}}=\overset{︷}{\underset{\infty }{T}}+\overset{︷}{\underset{0}{T}}x^{-1}$ is the changeable temperature at the surface, the free stream is maintained at $\overset{︷}{\underset{\infty }{T}}$ and $\overset{︷}{\underset{\infty }{T}}<\overset{︷}{\underset{w}{T}}$ , the fluid moves uniformly at the free stream with velocity $U_{\infty }$ , the velocity and thermal slip factors are denoted by L and K , respectively and the no-slip condition of MWCNTs-PG is recovered by setting L=0=K .

The supporting similarity equations for the problem are given by the following mathematical relations:

$$\beta \left(\eta \right)=\frac{\overset{︷}{T}-\overset{︷}{\underset{\infty }{T}}}{\overset{︷}{\underset{w}{T}}-\overset{︷}{\underset{\infty }{T}}},\eta =\sqrt{\frac{U_{\infty }}{x\nu_{f}}}y\;\text{and}\;\overset{︷}{\Psi }=\sqrt{\nu_{f}xU_{\infty }}F\left(\eta \right)$$

The velocity components $\overset{\vee }{u}$ and $\overset{\vee }{v}$ can be achieved through the rule $\overset{\vee }{u}=\frac{\partial \overset{︷}{\Psi }}{\partial y}$ and $\overset{\vee }{v}=-\frac{\partial \overset{︷}{\Psi }}{\partial x}$

Finally, after plugging the defined rules in the constitutive MWCNTs-PG model, the following self-similar version is achieved:

$$\frac{1}{{\left(1-\phi \right)}^{2.5}\left(\pi \left\{\left(1-\phi \right)+\frac{\phi {\overset{︷}{\rho }}_{MWCNTs}}{{\overset{︷}{\rho }}_{PG}}\right\}\right)}F^{\prime \prime \prime }+\frac{1}{\left\{\left(1-\phi \right)+\frac{\phi {\overset{︷}{\rho }}_{MWCNTs}}{{\overset{︷}{\rho }}_{PG}}\right\}}\lambda \beta Cos\;\gamma +0.5FF^{\prime \prime }=0,$$ (4)

$$\begin{array}{l} \left(1+\frac{Rd}{\left\{\frac{\left(1-\phi \right)+\frac{\frac{2\phi \overset{︷}{\underset{MWCNTs}{k}}}{\overset{︷}{\underset{MWCNTs}{k}}-\overset{︷}{\underset{PG}{k}}}\ln\left(\overset{︷}{\underset{MWCNTs}{k}}+\overset{︷}{\underset{PG}{k}}\right)}{2\overset{︷}{\underset{PG}{k}}}}{\left(1-\phi \right)+\frac{\frac{2\phi \overset{︷}{\underset{PG}{k}}}{\overset{︷}{\underset{MWCNTs}{k}}-\overset{︷}{\underset{PG}{k}}}\ln\left(\overset{︷}{\underset{MWCNTs}{k}}+\overset{︷}{\underset{PG}{k}}\right)}{2\overset{︷}{\underset{PG}{k}}}}\right\}}\right)\beta^{\prime \prime }+\\ \frac{\left(\frac{1}{{\left(1-\phi \right)}^{2.5}}\mathrm{Pr}Ec{F^{\prime \prime }}^{2}+0.5\left\{\left(1-\phi \right)+\frac{\phi {\left(\overset{︷}{\rho c_{p}}\right)}_{MWCNTs}}{{\left(\overset{︷}{\rho c_{p}}\right)}_{PG}}\right\}\mathrm{Pr}F\beta^{\prime }\right)}{\left\{\frac{\left(1-\phi \right)+\frac{\frac{2\phi \overset{︷}{\underset{MWCNTs}{k}}}{\overset{︷}{\underset{MWCNTs}{k}}-\overset{︷}{\underset{PG}{k}}}\ln\left(\overset{︷}{\underset{MWCNTs}{k}}+\overset{︷}{\underset{PG}{k}}\right)}{2\overset{︷}{\underset{PG}{k}}}}{\left(1-\phi \right)+\frac{\frac{2\phi \overset{︷}{\underset{PG}{k}}}{\overset{︷}{\underset{MWCNTs}{k}}-\overset{︷}{\underset{PG}{k}}}\ln\left(\overset{︷}{\underset{MWCNTs}{k}}+\overset{︷}{\underset{PG}{k}}\right)}{2\overset{︷}{\underset{PG}{k}}}}\right\}}=0 \end{array}$$ (5)

The new version of the flow conditions is achieved as:

$$F\left(\eta_{=0}\right)=S,\;F^{\prime }\left(\eta_{=0}\right)=F^{\prime \prime }\left(\eta_{=0}\right)\delta ,\;\beta \left(\eta_{=0}\right)=1+\beta^{*}\beta \prime \left(\eta_{=0}\right)$$

$$F^{\prime }\left(\eta_{\infty }\right)=1,\;\beta \left(\eta_{\infty }\right)=0$$

The governing flow parameters are defined by the following relations in Table 3:

**TABLE 3:** The governing flow quantities.

Parameter	Name	Mathematical form
λ	Mixed convection parameter	$\frac{g\beta^{*}\overset{︷}{\underset{0}{T}}}{U_{\infty }}$
Ec	Eckert number	$\frac{U_{\infty }^{2}}{{\left(c_{p}\right)}_{PG}\left(\overset{︷}{\underset{w}{T}}-\overset{︷}{\underset{\infty }{T}}\right)\;}$
Pr	Prandtl number	$\frac{\mu_{PG}{\left(c_{p}\right)}_{PG}}{k_{PG}}$

## Mathematical Analysis of MWCNTs-PG Model

The flow situations in various engineering systems and fluid mechanics are governed by a coupled nonlinear nature mathematical model. It is imperative to explore the solution of this model because the only way to analyze the dynamics of the model against the involved parameters is the solution of that model. Therefore, we need to obtain the model solution either in closed form, series form, or numerically. Such models cannot be tackled in the form of closed solutions due to high nonlinearity. However, numerical schemes are reliable and efficient for such models. Thus, a numerical scheme along with the coupling of shooting techniques (Ahmed et al., 2019; Khan et al., 2021) implemented. Primarily, this technique is based on the reduction of a higher-order nonlinear model into a system of first-order *via* some sort of transformation. For the particular model, the following transformations are plugged into the higher-order nonlinear model:

$$\begin{array}{l} F\left(\eta \right)={\overset{︷}{A}}_{1}^{*},F^{\prime }\left(\eta \right)={\overset{︷}{A}}_{2}^{*},F^{\prime \prime }\left(\eta \right)=\overset{︷}{{\overset{\vee }{A}}_{3}^{*}},F^{\prime \prime \prime }\left(\eta \right)={\overset{︷}{A}}_{3}^{*\prime },\\ \beta \left(\eta \right)={\overset{︷}{ℬ}}_{4}^{*},\beta \prime \left(\eta \right)={\overset{︷}{ℬ}}_{5}^{*},\beta \prime \prime \left(\eta \right)={\overset{︷}{ℬ}}_{5}^{*\prime } \end{array}$$ (6)

In accordance with these transformations, the higher-order model is transformed in the version:

$$\frac{1}{{\left(1-\phi \right)}^{2.5}\left(\left\{\left(1-\phi \right)+\frac{\phi {\overset{︷}{\rho }}_{MWCNTs}}{{\overset{︷}{\rho }}_{PG}}\right\}\right)}{\overset{︷}{A}}_{3}^{{*}^{\prime }}+\frac{1}{\left\{\left(1-\phi \right)+\frac{\phi {\overset{︷}{\rho }}_{MWCNTs}}{{\overset{︷}{\rho }}_{PG}}\right\}}\lambda {\overset{︷}{ℬ}}_{4}^{*}Cos\;\gamma +0.5{\overset{︷}{A}}_{1}^{*}\overset{︷}{{\overset{\vee }{A}}_{3}^{*}}=0$$

$$\begin{array}{l} \left(1+\frac{Rd}{\left\{\frac{\left(1-\phi \right)+\frac{\frac{2\phi \overset{︷}{\underset{MWCNTs}{k}}}{\overset{︷}{\underset{MWCNTs}{k}}-\overset{︷}{\underset{PG}{k}}}\ln\left(\overset{︷}{\underset{MWCNTs}{k}}+\overset{︷}{\underset{PG}{k}}\right)}{2\overset{︷}{\underset{PG}{k}}}}{\left(1-\phi \right)+\frac{\frac{2\phi \overset{︷}{\underset{PG}{k}}}{\overset{︷}{\underset{MWCNTs}{k}}-\overset{︷}{\underset{PG}{k}}}\ln\left(\overset{︷}{\underset{MWCNTs}{k}}+\overset{︷}{\underset{PG}{k}}\right)}{2\overset{︷}{\underset{PG}{k}}}}\right\}}\right){\overset{︷}{ℬ}}_{5}^{*\prime }+\\ \frac{\left(\frac{1}{{\left(1-\phi \right)}^{2.5}}\mathrm{Pr}Ec\overset{2}{\overset{︷}{{\overset{\vee }{A}}_{3}^{*}}}+0.5\left\{\left(1-\phi \right)+\frac{\phi {\left(\overset{︷}{\rho c_{p}}\right)}_{MWCNTs}}{{\left(\overset{︷}{\rho c_{p}}\right)}_{PG}}\right\}\mathrm{Pr}{\overset{︷}{A}}_{1}^{*}{\overset{︷}{ℬ}}_{5}^{*}\right)}{\left\{\frac{\left(1-\phi \right)+\frac{\frac{2\phi \overset{︷}{\underset{MWCNTs}{k}}}{\overset{︷}{\underset{MWCNTs}{k}}-\overset{︷}{\underset{PG}{k}}}\ln\left(\overset{︷}{\underset{MWCNTs}{k}}+\overset{︷}{\underset{PG}{k}}\right)}{2\overset{︷}{\underset{PG}{k}}}}{\left(1-\phi \right)+\frac{\frac{2\phi \overset{︷}{\underset{PG}{k}}}{\overset{︷}{\underset{MWCNTs}{k}}-\overset{︷}{\underset{PG}{k}}}\ln\left(\overset{︷}{\underset{MWCNTs}{k}}+\overset{︷}{\underset{PG}{k}}\right)}{2\overset{︷}{\underset{PG}{k}}}}\right\}}=0 \end{array}$$

The convergence of the technique is subject to the adjustment of $\eta_{\infty }$ and then the results for the dynamics of MWCNTs-PG were plotted by altering the governing parameters using MATHEMATICA 10.0.

## Graphical Results With Discussion

### MWCNTs-PG Motion Against δ and ϕ

This subsection presents the motion of MWCNTs-PG by increasing the strength of the velocity parameter and fraction factor of MWCNTs. The results plotted for buoyancy added (λ>0) , buoyancy opposed (λ<0) , and in the absence of combined convection, respectively.

**FIGURE 2:**
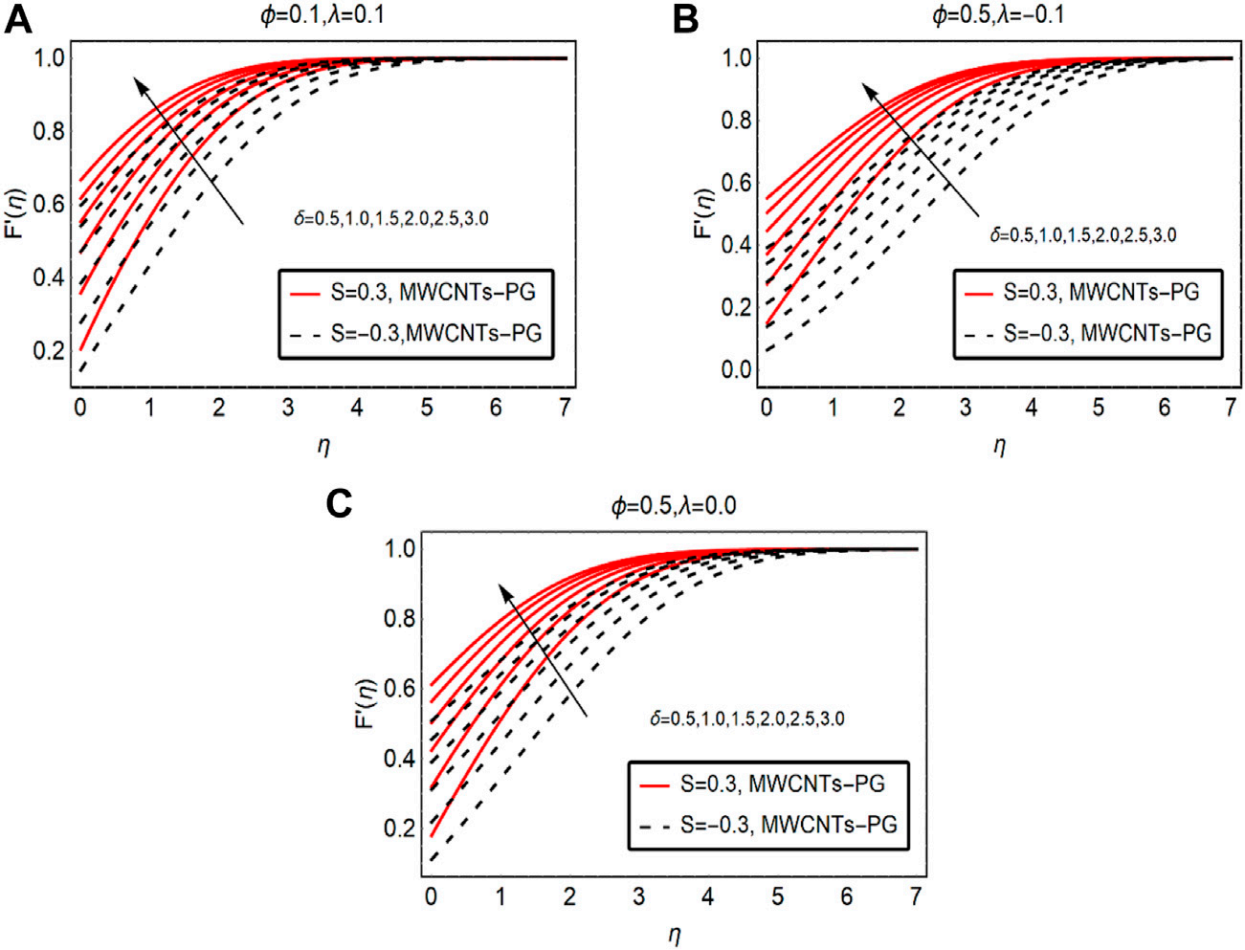
$F^{\prime }\left(\eta \right)$ against δ **(A)** λ>0 and **(B)** λ<0 and **(C)** λ=0.

Figure 2 captures the behavior of fluid motion against rising velocity slip parameter δ . The fluid motion is in direct proportion with the strength of the slip parameter. Near the inclined surface, the velocity abruptly rises, and then with time it reduces and then finally approaches the free stream velocity. Physically, at the surface vicinity, the slip effects are dominant and maximum variations in the velocity are examined. The force of friction between the fluid layer adjacent to the surface and the surface of the sheet is reduced due to the slip effects. As a result, the fluid particles move freely and abruptly over the surface. For buoyancy opposed flow, these variations are quite rapid and buoyancy assists flow scenarios. The momentum boundary layer region increases for buoyancy assisted flow of MWCNTs-PG.

The influences of suction and injection are also captured for MWCNTs-PG. Based on the results, the injection of MWCNTs-PG from the surface favors the movement of the fluid over the surface. Physically, injecting fluid exerted extra force on the fluid particles, which ultimately boosts the momentum of the particles. Due to increasing momentum, the fluid motion rises whereas, in the case of suction, the motion is slow because the fluid particles are stuck at the surface, which slightly decreases the fluid momentum. Therefore, the fluid moves more slowly than when injected.

**FIGURE 3:**
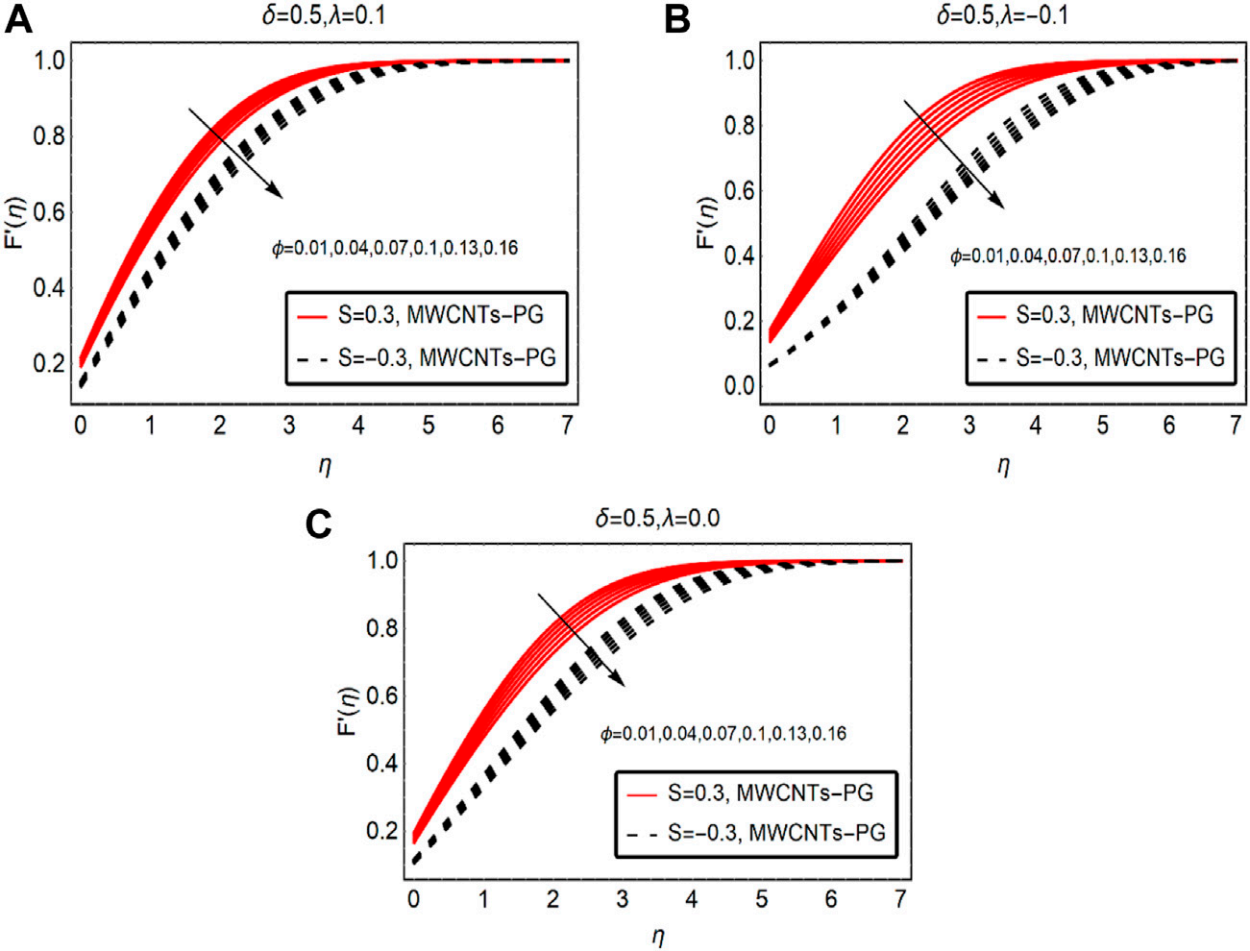
$F^{\prime }\left(\eta \right)$ against ϕ **(A)** λ>0 and **(B)** λ<0 and **(C)** λ=0.

Figure 3 examines the fluid motion by strengthening the fraction factor of MWCNTs. By increasing the strength of the fraction factor within the feasible domain, fluid motion is resisted. Physically, the mixture becomes denser due to the high volumetric fraction and intermolecular forces between the fluid particles becoming dominant. As a result, the fluid motion drops. The maximum drop in the motion is observed for buoyancy opposing flow and minimal decreasing variations are noted for buoyancy-assisted flow. These results are demonstrated in Figures 3A–C, respectively.

### MWCNTs-PG Thermal Distribution Against Ec and $\beta^{*}$

**FIGURE 4:**
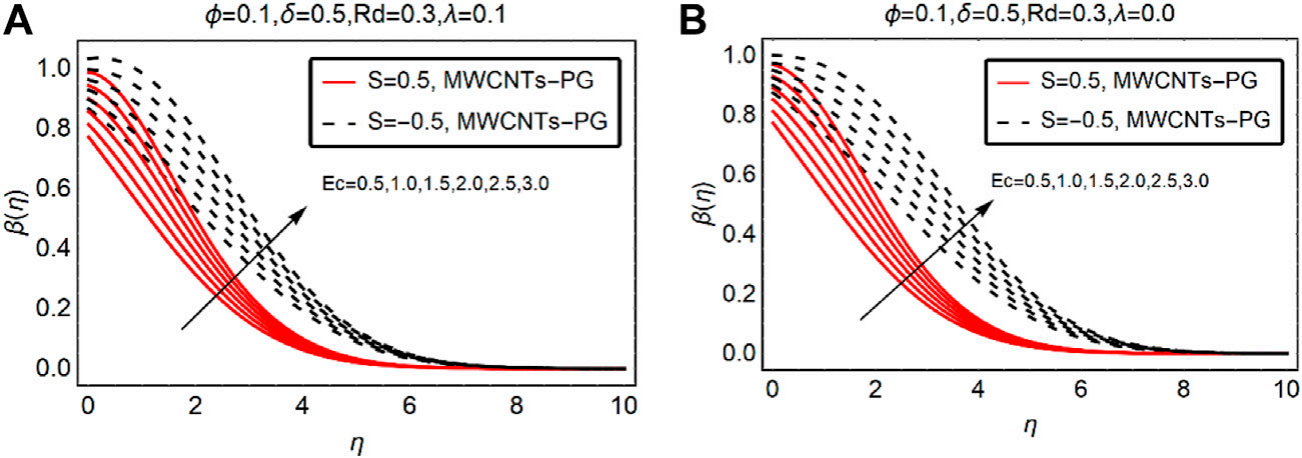
β(η) against Ec **(A)** λ>0 and **(B)** λ=0.

**FIGURE 5:**
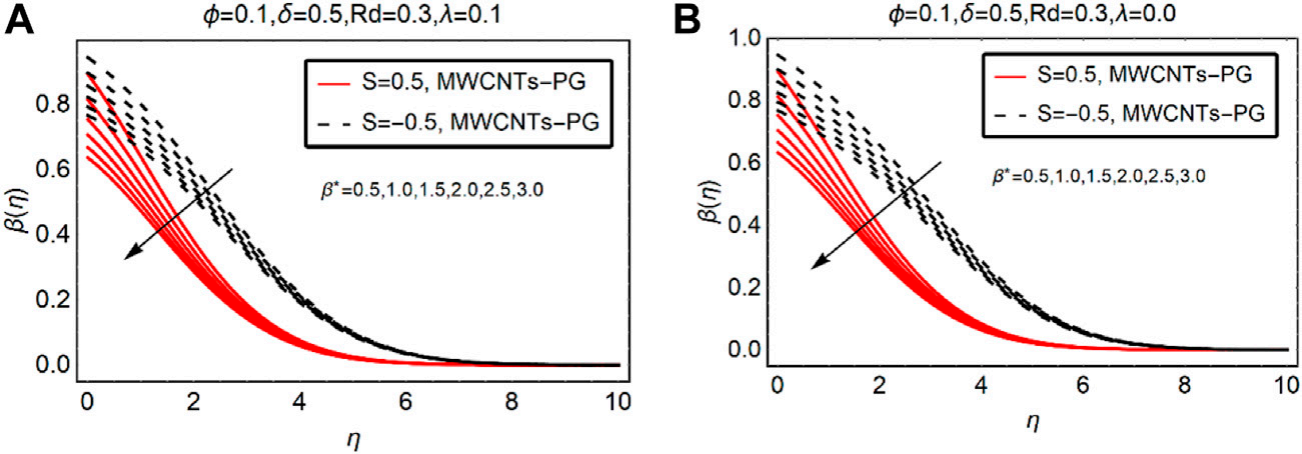
β(η) against $\beta^{*}$ **(A)** λ>0 and **(B)** λ=0.

The viscous dissipation and thermal slip parameters significantly alter the fluid thermal behavior over the desired domain. Therefore, Figures 4, 5 are decorated to analyze the temperature trends of MWCNTs-PG against Ec and $\beta^{*}$ . The temperature of MWCNTs-PG enhances for more dissipative fluid. The fluid temperature prominently rises near the surface for both suction and injection of the fluid. Physically, the internal energy of the fluid particles enhances due to more dissipation effects and it leads to an increment in the thermal field. The temperature far from the surface obeys the ambient temperature condition and finally vanishes by showing its asymptotic behavior.

For injection of the fluid, the temperature rises more rapidly than suction. Physically, injecting fluid transfers energy to the fluid particles and the internal energy of MWCNTs-PG boosts. This leads to an increment in the thermal profile β(η) . The temperature trends against the thermal slip parameter are demonstrated in Figure 5. It is noteworthy that, in the presence of combined convection effects, the temperature drops near the surface by strengthening the thermal slip parameter.

### MWCNTs-PG Thermal Distribution Against δ and Rd

**FIGURE 6:**
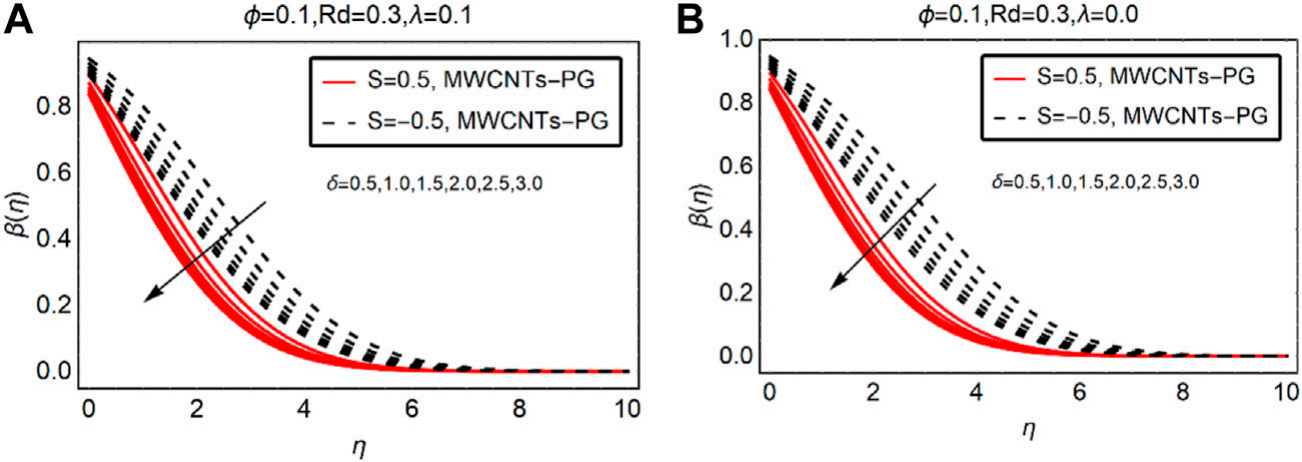
β(η) against δ **(A)** λ>0 and **(B)** λ=0.

**FIGURE 7:**
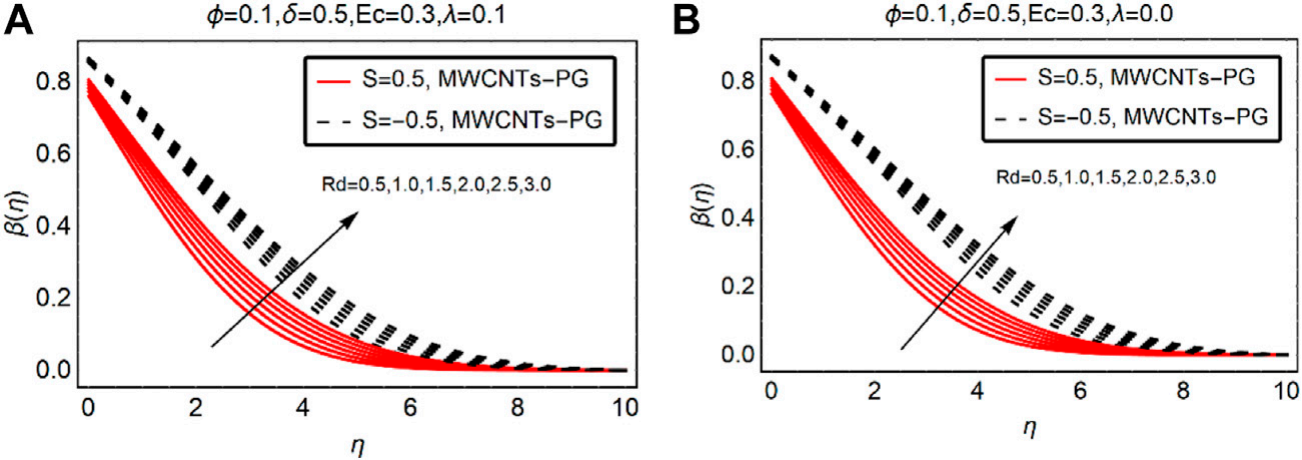
β(η) against Rd **(A)** λ>0 and **(B)** λ=0.

The temperature alterations due to the velocity and thermal radiation parameters are elaborated in Figures 6, 7, respectively. From the analysis of Figure 6, the resistive behavior of δ on the temperature field is observed. The temperature rapidly drops for the suction case. Physically, the fluid particles stick to the surface due to the suction of the fluid and intermolecular forces between them becoming more dominant. This drops the momentum of the fluid and ultimately collisions between the particles become slow, which leads to a decrement in temperature β(η) . These effects are pictured in Figures 6A,B for buoyancy assisted and without convection, respectively.

Figure 7 elaborates on the influence of thermal radiations on the temperature performance of MWCNTs-PG. The imposed thermal radiations favor the temperature of the nanofluid. Physically, the internal energy of MWCNTs-PG rises due to thermal radiations, and in turn the temperature rises. For suction of the fluid, the rise in the temperature is slower than the injecting fluid. The injecting fluid provides extra energy to the fluid particles due to the existence of thermal radiations, which significantly increase the temperature.

### Shear Stresses and Local Energy Storage Ability

**FIGURE 8:**
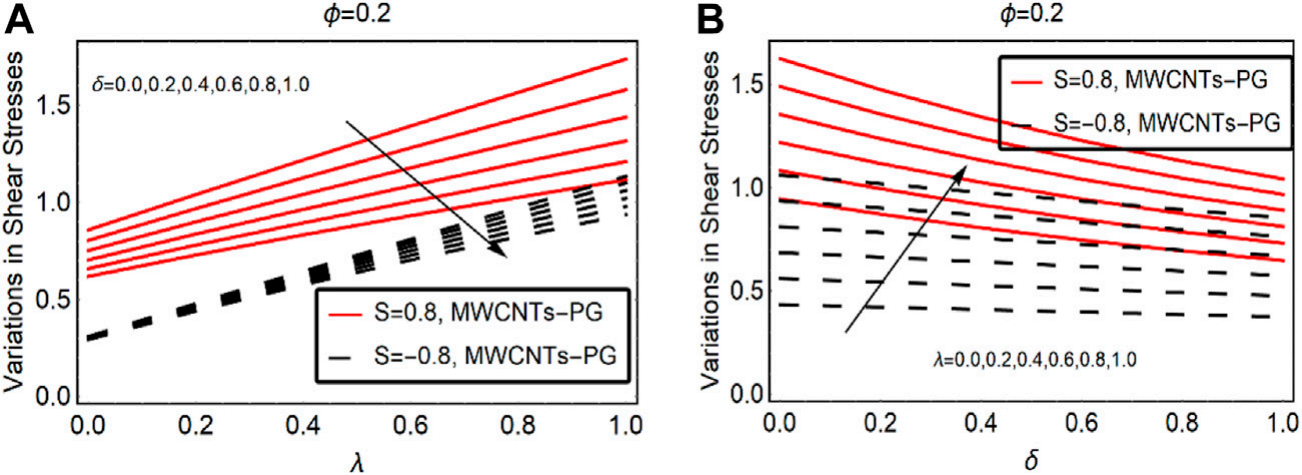
The shear stresses against **(A)** δ and **(B)** λ.

The trends in surface shear stress due to altering the velocity slip and combined convection parameter are elaborated in Figure 8. The shear stresses at the surface decline quickly against δ . However, for injection cases, rapid decrement in the shear stresses was investigated. The reason for this decrease is that fluid particles left the surface due to the injecting fluid as a result transformation of the surface stresses drops. On the other hand, combined convection favors the transformation of the shear stresses at the surface. This behavior is outlined in Figures 8A, B, respectively.

**FIGURE 9:**
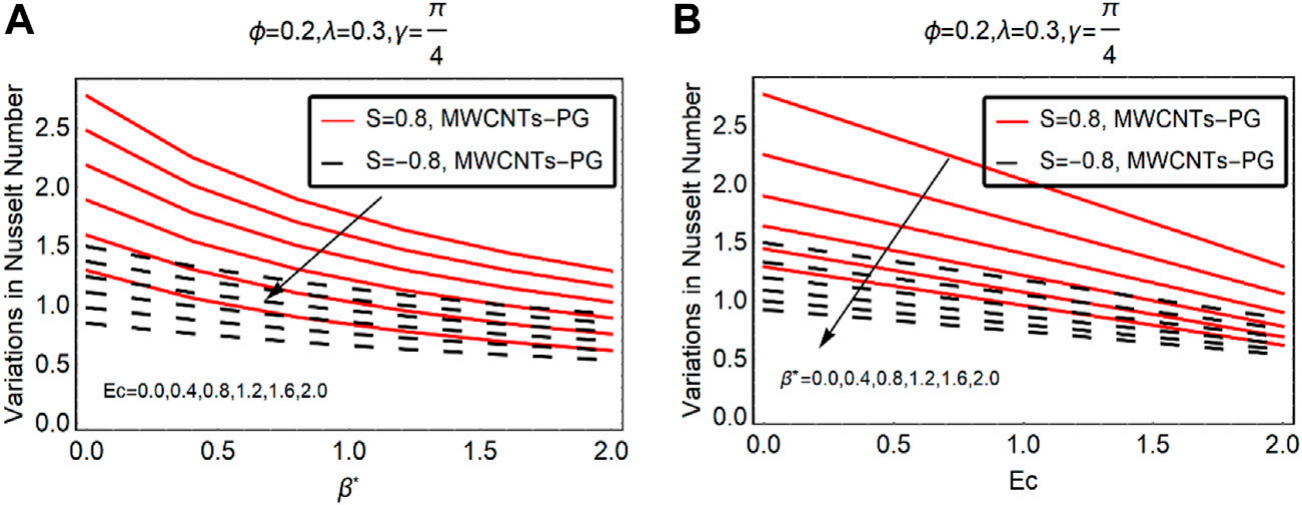
The local thermal performance rate against **(A)** Ec and **(B)** $\beta^{*}.$

**FIGURE 10:**
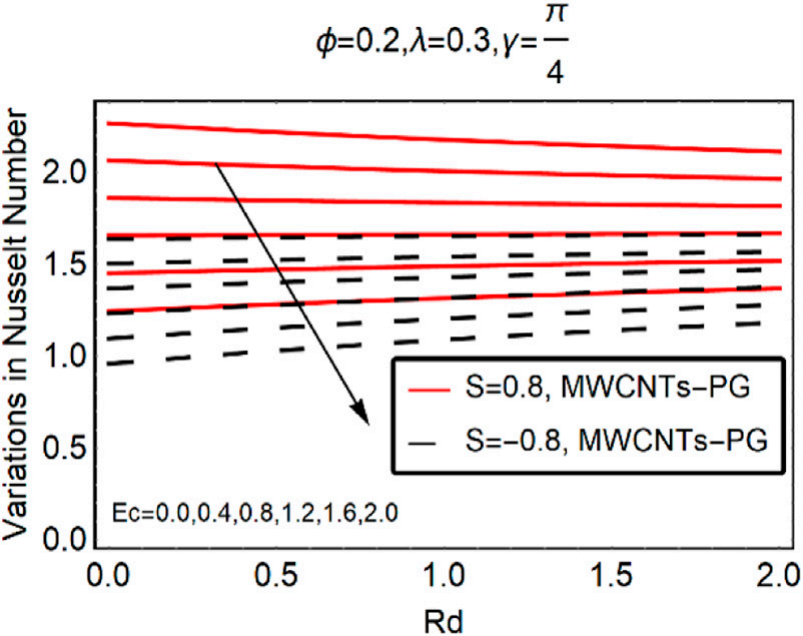
The local thermal performance rate against Ec and Rd.

The local thermal performance of MWCNTs-PG against Ec, $\beta^{*}$ and thermal radiation over Ec are demonstrated in Figures 9, 10, respectively. On inspection of the results, the local thermal performance rate in the particular nanoliquid drops Ec and $\beta^{*}$ . By enhancing the strength of these parameters, the decrement becomes slowed down. Similar trends in the local heat transport mechanism are highlighted in Figure 10.

Table 4 provides a summary of the results against the varying flow quantities. It covers all the questions of the conducted research.

**TABLE 4:** Summary of the dynamics of MWCNTs-PG against the pertinent flow parameters.

Parameters	Velocity		Temperature		Local Energy storage		Shear stresses	
	S>0	S<0	S>0	S<0	S>0	S<0	S>0	S<0
δ	Increases	Increases	Decreases	Decreases	—	—	Decreases	Decreases
ϕ	Decreases	Decreases	—	—	—	—	—	—
Ec	—	—	Increases	Increases	Decreases	Decreases	—	—
$\beta^{*}$	—	—	Decreases	Decreases	Decreases	Decreases	—	—
Rd	—	—	Increases	Increases	—	—	—	—
λ	—	—	—	—	—	—	Increases	Increases

## Concluding Remarks

This study undertook a numerical investigation of thermal storage in MWCNTs-PG over an inclined geometry. The influences of combined convection, thermal radiation, velocity, and thermal slip on the dynamics of the nanoliquid were demonstrated against the governing parameters. We inspected buoyancy assisted and buoyancy opposed. In the absence of combined convection, the nanofluid motion enhances both suction and injection of the fluid. However, the fraction factor of MWCNTs resists the nanoliquid motion. the stronger dissipation and thermal radiations effects improve the energy storage capacity of MWCNTs-PG. Decreasing trends were investigated against the velocity and thermal slip parameters. It can be concluded that the nanoliquid has high energy storage abilities and it would be beneficial for industrial and engineering applications.

## Data Availability

The data supporting the study is available within the article.

## References

[B1] Ahmed N. Khan U. U. Mohyud-Din S. T. (2017). Influence of thermal Radiation and Viscous Dissipation on Squeezed Flow of Water between Riga Plates Saturated with Carbon Nanotubes. Colloids Surf. A: Physicochemical Eng. Aspects 522, 389–398. 10.1016/j.colsurfa.2017.02.083

[B2] Ahmed N. Khan U. U. Mohyud-Din S. T. (2020). Modified Heat Transfer Flow Model for SWCNTs-H2o and MWCNTs-H2o over a Curved Stretchable Semi Infinite Region with thermal Jump and Velocity Slip: A Numerical Simulation. Physica A: Stat. Mech. its Appl. 545, 123431. 10.1016/j.physa.2019.123431

[B3] Ahmed N. Tassaddiq A. Alabdan R. Adnan U. Khan U. Noor S. (2019). Applications of Nanofluids for the Thermal Enhancement in Radiative and Dissipative Flow over a Wedge. Appl. Sci. 9 (10), 1976. 10.3390/app9101976

[B4] Bej S. Das R. Mondal A. Saha R. Sarkar K. Banerjee P. (2022). Knoevenagel Condensation Triggered Synthesis of Dual-Channel Oxene Based Chemosensor: Discriminative Spectrophotometric Recognition of F−, CN– and HSO4− with Breast Cancer Cell Imaging, Real Sample Analysis and Molecular Keypad Lock Applications. Spectrochimica Acta A: Mol. Biomol. Spectrosc. 273, 120989. 10.1016/j.saa.2022.120989 35183856

[B5] Bej S. Mandal S. Mondal A. Pal T. K. Banerjee P. (2021). Solvothermal Synthesis of High-Performance D10-MOFs with Hydrogel Membranes @ "Turn-On" Monitoring of Formaldehyde in Solution and Vapor Phase. ACS Appl. Mater. Inter. 13 (21), 25153–25163. 10.1021/acsami.1c05998 34011156

[B6] Benos L. T. Karvelas E. G. Sarris I. E. (2019). Crucial Effect of Aggregations in CNT-Water Nanofluid Magnetohydrodynamic Natural Convection. Therm. Sci. Eng. Prog. 11, 263–271. 10.1016/j.tsep.2019.04.007

[B7] Bhattacharyya K. Mukhopadhyay S. Layek G. C. (2013). Similarity Solution of Mixed Convective Boundary Layer Slip Flow over a Vertical Plate. Ain Shams Eng. J. 4, 299–305. 10.1016/j.asej.2012.09.003

[B8] Das S. Jana R. N. Makinde O. D. (2015). Magnetohydrodynamic Mixed Convective Slip Flow over an Inclined Porous Plate with Viscous Dissipation and Joule Heating. Alexandria Eng. J. 54, 251–261. 10.1016/j.aej.2015.03.003

[B9] Gireesha B. J. Kumar K. G. Ramesh G. K. Prasannakumara B. C. (2018). Nonlinear Convective Heat and Mass Transfer of Oldroyd-B Nanofluid over a Stretching Sheet in the Presence of Uniform Heat Source/sink. Results Phys. 9, 1555–1563. 10.1016/j.rinp.2018.04.006

[B10] Hassani S. Taylor R. A. Mekhilef S. Saidur R. (2016). A cascade Nanofluid-Based PV/T System with Optimized Optical and thermal Properties. Energy 112, 963–975. 10.1016/j.energy.2016.06.142

[B11] Hayat T. Khan S. A. Ijaz Khan M. Momani S. Alsaedi A. (2020). Cattaneo-Christov (CC) Heat Flux Model for Nanomaterial Stagnation point Flow of Oldroyd-B Fluid. Computer Methods Programs Biomed. 187, 105247. 10.1016/j.cmpb.2019.105247 31812885

[B12] Hayat T. Qayyum S. Alsaedi A. Ahmad B. (2020). Entropy Generation Minimization: Darcy-Forchheimer Nanofluid Flow Due to Curved Stretching Sheet with Partial Slip. Int. Commun. Heat Mass Transfer 111, 104445. 10.1016/j.icheatmasstransfer.2019.104445

[B13] Khan M. Malik M. Y. Salahuddin T. Hussian A. (2018). Heat and Mass Transfer of Williamson Nanofluid Flow Yield by an Inclined Lorentz Force over a Nonlinear Stretching Sheet. Results Phys. 8, 862–868. 10.1016/j.rinp.2018.01.005

[B14] Khan U. Ahmed N. Khan I. Baleanu D. Nisar K. S. (2021). Al2O3 and Gamma Al2O3 Nanomaterials Based Nanofluid Models with Surface Diffusion: Applications for thermal Performance in Multiple Engineering Systems and Industries. Comput. Mater. Continua 66 (2), 1563–1576. 10.32604/cmc.2020.012326

[B15] Mukhopadhyay S. Chandra Mandal I. (2015). Magnetohydrodynamic (MHD) Mixed Convection Slip Flow and Heat Transfer over a Vertical Porous Plate. Eng. Sci. Technol. Int. J. 18, 98–105. 10.1016/j.jestch.2014.10.001

[B16] Punith Gowda R. J. Al-Mubaddel F. S. Naveen Kumar R. Prasannakumara B. C. Issakhov A. Rahimi-Gorji M. (2021). Computational Modelling of Nanofluid Flow over a Curved Stretching Sheet Using Koo-Kleinstreuer and Li (KKL) Correlation and Modified Fourier Heat Flux Model. Chaos, Solitons & Fractals 145, 110774. 10.1016/j.chaos.2021.110774

[B17] Reddy P. S. Sreedevi P. (2021). Flow and Heat Transfer Analysis of Carbon Nanotubes Based Nanofluid Flow inside a Cavity with Modified Fourier Heat Flux. Phys. Scr. 96, 055215. 10.1088/1402-4896/abe90f

[B18] Reddy P. S. Sreedevi P. Reddy V. N. (2022). Entropy Generation and Heat Transfer Analysis of Magnetic Nanofluid Flow inside a Square Cavity Filled with Carbon Nanotubes. Chem. Thermodynamics Therm. Anal. 6, 100045. 10.1016/j.ctta.2022.100045

[B19] Shaiq S. Maraj E. N. (2019). Role of the Induced Magnetic Field on Dispersed CNTs in Propylene Glycol Transportation toward a Curved Surface. Arab J. Sci. Eng. 44, 7515–7528. 10.1007/s13369-019-03828-4

[B20] Uddin M. J. Kabir M. N. Bég O. A. (2016). Computational Investigation of Stefan Blowing and Multiple-Slip Effects on Buoyancy-Driven Bioconvection Nanofluid Flow with Microorganisms. Int. J. Heat Mass Transfer 95, 116–130. 10.1016/j.ijheatmasstransfer.2015.11.015

[B21] Waini I. Ishak A. Pop I. (2020). Transpiration Effects on Hybrid Nanofluid Flow and Heat Transfer over a Stretching/shrinking Sheet with Uniform Shear Flow. Alexandria Eng. J. 59 (1), 91–99. 10.1016/j.aej.2019.12.010

[B22] Yasmeen T. Hayat T. Khan M. I. Imtiaz M. Alsaedi A. (2016). Ferrofluid Flow by a Stretched Surface in the Presence of Magnetic Dipole and Homogeneous-Heterogeneous Reactions. J. Mol. Liquids 223, 1000–1005. 10.1016/j.molliq.2016.09.028

[B23] Yazdanifard F. Ameri M. Taylor R. A. (2020). Numerical Modeling of a Concentrated Photovoltaic/thermal System Which Utilizes a PCM and Nanofluid Spectral Splitting. Energ. Convers. Manag. 215, 112927. 10.1016/j.enconman.2020.112927

